# Breast Cancer Survivors Undergoing Endocrine Therapy Have a Worrying Risk Factor Profile for Cardiovascular Diseases

**DOI:** 10.3390/nu13041114

**Published:** 2021-03-29

**Authors:** Fernanda S. Mazzutti, Isis D. D. Custódio, Mariana T. M. Lima, Kamila P. de Carvalho, Taísa S. S. Pereira, Maria del C. B. Molina, Paula P. L. Canto, Carlos E. Paiva, Yara C. de P. Maia

**Affiliations:** 1 Molecular Biology and Nutrition Research Group, School of Medicine, Federal University of Uberlandia, Uberlandia 38405-320, Brazil; fernandamazzutti@hotmail.com (F.S.M.); isisdanyelle@yahoo.com.br (I.D.D.C.); tmmariana@hotmail.com (M.T.M.L.); kamila@ufu.br (K.P.d.C.); 2 Nutrition Science, Department of Health Sciences, University of the Americas Puebla, Cholula 72810, Mexico; taisa.sabrina@hotmail.com; 3 Graduate Program in Nutrition and Health, Federal University of Espirito Santo, Vitoria 29047-105, Brazil; mdmolina@uol.com.br; 4 Nutrition Course, Federal University of Espirito Santo, Vitoria 29047-105, Brazil; 5 Department of Clinical Oncology, Clinic’s Hospital, Federal University of Uberlandia, Uberlandia 38405-320, Brazil; pplajolo@uol.com.br; 6 Department of Clinical Oncology, Graduate Program in Oncology, Barretos 14784-400, Brazil; drcarlosnap@gmail.com; 7 Palliative Care and Quality of Life Research Group (GPQual), Pio XII Foundation-Barretos Cancer Hospital, Barretos 14784-400, Brazil; 8 Nutrition Course, Medical Faculty, Federal University of Uberlandia, Uberlandia 38405-320, Brazil

**Keywords:** cancer survivors, breast neoplasms, cardiovascular diseases, endocrine therapy, food consumption, body composition, anthropometry, biomarkers

## Abstract

The increased risk for cardiovascular diseases (CVDs) in breast cancer survivors has been widely discussed in the literature and occurs due to the cardiotoxicity of antineoplastic treatments, and also to the common risk factors between these diseases. Thus, the objective of our study was to evaluate, prospectively, the number of risk factors (NRF) for CVDs in women during endocrine therapy, and to associate the NRF with C reactive protein (CRP) and phase angle (PhA). The following risk factors for CVD were evaluated at three times: anthracycline chemotherapy, radiotherapy, comorbidities, inadequate diet, overweight, abdominal adiposity, alcoholism, smoking, physical inactivity and altered lipid profile. There was inadequacy in the most components of the Brazilian Healthy Eating Index—Revised and inadequate consumption of various types of fats and fibers. Most women in this study presented excessive abdominal fat and overweight, but these parameters have not changed over time (*p* < 0.005). Moreover, a high frequency of systemic arterial hypertension and physical inactivity was observed. The average NRF for CVDs was above ten, at the three evaluation times. Women with higher NRF had higher levels of CRP (*p* = 0.003), a predictor of cardiovascular risk, however, there was no significance with PhA (*p* = 0.256). Thus, intervention is needed to improve lifestyle.

## 1. Introduction

Breast cancer (BC) is the most common type of cancer in women [1]. Improvements in BC treatment have led to increasing chances of cure in approximately 70% to 80% of patients with early disease [2]. Despite improvements, BC survivors have higher risk of mortality from cardiovascular diseases (CVDs) when compared to those not diagnosed with the disease [3]. Such conditions may occur due to the higher prevalence of risk factors for CVDs, such as dyslipidemia, abdominal adiposity, systemic arterial hypertension (SAH) and diabetes mellitus (DM) [4,5]. In addition, antineoplastic treatments, such as chemotherapy (CT) with anthracycline, radiotherapy and endocrine therapy, can lead to cardiotoxicity [6,7].

Endocrine therapy is prescribed, aiming to reduce BC recurrence and mortality [8,9]. Among the drugs used in this treatment, aromatase inhibitors (AIs) have high efficacy in women in the postmenopausal stage [10,11], however, they are associated with increased risk of vascular disease, myocardial infarction and angina [12]. One possible explanation is that the drug reduces estrogen levels, and these hormones are related to cardiovascular protection [13]. A prospective study has shown that 80% of women with prescriptions for this therapy have a predicted risk of CVDs in ten years equal to or higher than the risk of recurrence of BC [14].

The adoption of a better-quality diet is associated with a reduction in the incidence of and mortality from BC [15] and CVDs [16,17]. Similarly, the importance of body weight control is emphasized, with obesity and abdominal adiposity being associated with a higher risk of CVDs in BC survivors [18]. Physical activity, in turn, reduces risk factors for CVDs, such as changes in systolic blood pressure and excess body weight [19]. According to evidence from the Brazilian Society of Cardiology, any level of physical activity can reduce cardiovascular risk [20]. In addition to contributing to lower cardiovascular risk, the practices of physical activity and body weight control, as well as healthy food consumption, contribute positively to the best prognosis of BC [21]. Moreover, regarding the serum lipid profile, higher levels of total cholesterol and low-density lipoprotein (LDL) and/or reduction of high-density lipoprotein (HDL) are associated with increased atherosclerosis, stroke and heart attack [22].

Light or moderate alcohol consumption appears to have a positive impact on cardiovascular health in the general population [23], and in women with BC [24]. However, excessive alcohol consumption leads to increased cardiovascular risk, and consumption of no more than one dose of alcohol per day is recommended [23]. Smoking is also an important factor that can increase the risk of almost all types of CVDs [25], and is associated with the worst atherosclerosis subclinical measures (carotid intima–media thickness, ankle–brachial index, coronary artery calcium score) and with higher levels of C-reactive protein (CRP) [26]. CRP is an acute-phase protein which acts as a marker and regulator of inflammatory and infectious processes [27], and it is considered a strong predictor of cardiovascular events [28]. Similarly, phase angle (PhA), which reflects the integrity and the cellular function [29], is also associated with cardiovascular events [30]. The CRP levels and reduced PhA in the elderly are associated with increased cardiovascular risk [28,30]. Furthermore, these measures are indicative of poor prognosis of BC [31,32].

Due to the large number of CVD deaths among BC survivors, we hypothesize that these women present several CVD risks that predispose them to the disease. Besides that, the greater number of risk factors (NRF) may be related to markers such as higher CRP and lower PhA. Thus, this prospective study aimed to assess the presence of risk factors for CVDs and their change over time in BC survivors undergoing endocrine therapy, associating the NRF for CVDs with CRP and PhA.

## 2. Materials and Methods

### 2.1. Design and Ethics

A prospective study carried out from January 2016 to August 2018 with BC survivors undergoing endocrine therapy with AIs at the Clinical Hospital of the Federal University of Uberlandia (HC/UFU). The follow-up time was 24 months, and the evaluations were carried out in three assessments, denoted T0, initial follow-up period; T1, intermediate follow-up period, 12 months after T0; and T2, final follow-up period (T2), 24 months after T0.

### 2.2. Sample Size and Eligibility Criteria

**Figure 1:**
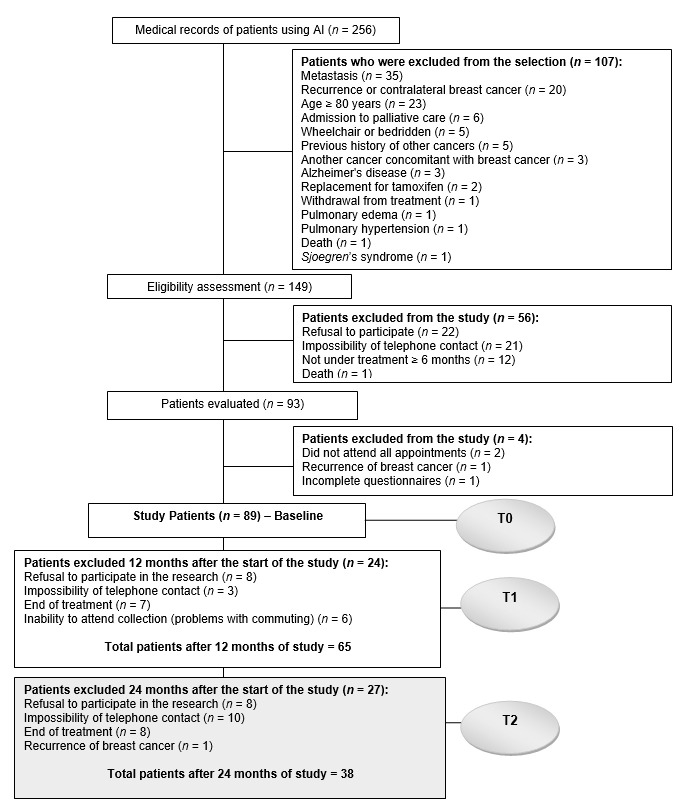
Diagram reporting the number of women recruited and selected in the study.

The recruitment of participants and exclusion criteria are described in Figure 1. Through the non-probabilistic sampling for convenience, women were recruited at the beginning, middle and end of the AI treatment. To avoid selection bias, the participants were listed consecutively. To calculate the sample size of a group of individuals and three measurements, the software G * Power version 3.1 [33] was used. A total of 28 women were required at each time, based on an ANOVA repeated measures F-test with an intermediate effect size of 0.25, an alpha level of 0.05 and 80% power.

### 2.3. Clinical, Sociodemographic and Lifestyle Data

In order to characterize the population through the analysis of medical records (T0), data on the type of surgery, radiotherapy and previous CT, tumor subtype, clinical stage, histological grade, positivity of hormone receptors, molecular subtype and duration of AIs were obtained. Anthracycline CT and radiotherapy were counted as cardiovascular risk factors.

Through interviews at T0, T1 and T2, personal, comorbidity and socioeconomic data were obtained, such as age, education, income and self-reported race and presence or absence of self-reported DM and SAH. Patients with SAH used medications such as diuretics (hydrochlorothiazide, chlortalidone or furosemide), beta-blockers (atenolol, bisoprolol or propranolol), calcium channel antagonists (nifedipine or anlodipine), angiotensin-converting enzyme inhibitors (captopril or enalapril), angiotensin II receptor antagonists (losartan or diovan), direct vasodilators (hydralazine hydrochloride) and/or sympatholytic drugs (methyldopa). Diabetic patients used medications such as biguanides (metformin), sulfonylureas (gliclazide) and insulin. Data on the history of CVDs were obtained through the analysis of medical records. Lifestyle data were also obtained, such as smoking (smokers or non-smokers), alcohol consumption (excessive or not) and physical activity (inactive, insufficiently active or active). Race and education were evaluated only at T0.

To be considered excessive, alcohol consumption should be at least 8 drinks per week, with each dose equivalent to approximately one can of beer or one glass of wine [34]. Other types of drinks were not consumed by the women in the present study. Regarding the practice of physical activity, women were classified according to Vigitel (2020) [35].

### 2.4. Anthropometric Measurement

Weight, height, waist circumference (WC) and hip circumference were measured. Specific protocol was used for all measures [36]. WC was classified according to the World Health Organization (WHO) [37], considering the following cut-off points: increased cardiovascular risk (>80 cm) and greatly increased cardiovascular risk (>80 cm). Body mass index (BMI) was calculated by dividing weight by height squared (kg/m^2^) and classified according to the recommended ranges for the adult population (age > 18 years and <60 years): without overweight up to 24.9 kg/m^2^ and overweight ≥ 25 kg/m^2^) [37], and elderly population (≥60 years old): without overweight up to 26.9 kg/m^2^ and overweight ≥ 27 kg/m^2^ [38]. The waist-to-hip ratio (WHR) and the waist-to-height ratio (WHtR) were obtained to assess the occurrence of abdominal obesity. They were classified, respectively, according to the WHO (risk of metabolic complications > 0.85) [37] and according to Ashwell and Hsieh (excess abdominal fat ≥ 0.5) [39]. Additionally, to assess abdominal fat, the conicity index (CI) was calculated [40]. The CI estimation uses variables such as weight, height and WC: $CI=\left(WC\;÷\left(0.109\;\sqrt{body}\;weight\;÷height\right)\right)$.

The horizontal tetra polar bioelectrical impedance (BIA) (Biodynamics, model 450) was used to evaluate body compartments and phase angle, according to the protocol by Cômodo and collaborators [41]. Participants were guided regarding the protocol of the test [42]. Women with changes in total body water were excluded from the analysis of body composition.

### 2.5. Biochemical Data

Venous blood was collected at the time of the interview after overnight fasting (up to 12 h) and under standard conditions [43]. Serum CRP concentrations were obtained at T1 and T2, as well as variables of the lipid profile. For these, the cut-off points were based on the guidelines by the Brazilian Society of Cardiology [20]: total cholesterol (risk ≥ 240 mg/dL), LDL-c (risk ≥ 160 mg/dL), HDL (risk < 40 mg/dL), non-HDL (risk ≥ 160 mg/ dL) and triglycerides (TGs) (mg/dL) (risk ≥ 200 mg/dL). CRP was considered as a continuous variable in the analyses. For dyslipidemia, patients used drugs of the statin class (simvastatins, artovastatin or rosuvastatin). Considering the anthropometric data together with the biochemical data, the following measures were obtained: visceral adiposity index (VAI) [44], which was calculated using WC (cm), BMI (kg/m^2^), TGs (mmol/L) and HDL (mmol/L) data: [VAI=(WC ÷36.58+(1.89 ×BMI))×(TG ÷0.81)×(1.52 ÷HDL)]; and lipid product accumulation (LAP) [45], calculated using WC (cm) and TGs (mmol/L) data: [LAP=(WC−58)×TG]. For these variables, there are no established cut-off points for cardiovascular risk.

### 2.6. Dietary Data

Food consumption information was collected through 24 h dietary recalls (24HRs). At each study session (T0, T1 and T2), three non-consecutive 24HRs were applied, one referring to a weekend day, to better reflect the participants’ eating habits, totaling nine 24HRs during the study. The first 24HR was carried out in person and the others via telephone, following the technique used in the Vigitel [35] study. From the 24HR, consumption was assessed quantitatively using the Nutrition Data System for Research (NDSR) software. The following nutrients were evaluated regarding cardiovascular risk: total fiber (risk 25 g, of which soluble fiber < 6 g), total fat (risk > 30% of the total caloric value—TCV), saturated fat (risk > 7% TCV), polyunsaturated fat (risk < 6 or >10% TCV), monounsaturated fat (risk < 15 or >20% TCV), trans fat (risk > 1% TCV) cholesterol (risk > 300 mg/day), sodium (risk > 2300 mg/day), omega 3 (risk < 1 g/day) and omega 6 and 3 ratio (risk > 5: 1). These cut-off points are included in the first guidelines on the consumption of fat and cardiovascular health [46], except for sodium, which was evaluated according to the recommendations by the Institute of Medicine [47]. Energy consumption (kcal), carbohydrates, sugars and proteins throughout the study were also evaluated, in order to obtain an overview of food consumption.

To verify food consumption over time, due to its intrinsic variability, the data were attenuated, that is, corrected by inter- and intra-individual variability, following the methodology by Nusser and collaborators [48], using the PC-Side software (Department of Statistics, Iowa State University, Ames, IA, USA), to obtain an estimate of the individual’s energy and nutrient consumption. Subsequently, as recommended by Willet, Howe and Kushi [49], in order to correct nutrient estimates, these were adjusted by a residual method by the mean energy consumption of the sample. However, to account for the cardiovascular risk of the individual from the consumption of nutrients, data that were only attenuated, without adjustments for energy, were used, so as not to underestimate the consumption.

#### Brazilian Healthy Eating Index—Revised

The Healthy Eating Index (HEI) [50] was adapted to Brazil, using the structure of HEI-2005 [51]. Subsequently, this index was revised in 2011 [52], developing the Brazilian Healthy Eating Index—Revised (BHEI-R), used in the present study for qualitative assessment of the diet. The BHEI-R includes the following food components: Total Fruit (fruit and natural fruit juices); Whole Fruit (excluding fruit juices); Total Vegetables (including legumes after reaching the maximum score for Meat, Eggs and Legumes); Dark Green and Orange Vegetables and Legumes (including legumes after reaching maximum scores for the Meat, Eggs and Legumes, and Total Vegetables groups); Total Cereals (including grains, roots and tubers); Whole Cereals; Milk and Dairy Products (including milk and milk derivatives, in addition to soy-based drinks); Meat, Eggs and Legumes; Oils (including mono- and polyunsaturated fats, oilseeds and fish fat); Saturated Fat; Sodium; and calories from trans and saturated fats, alcohol and added sugar (SoFAAS). For cooking oil, 5 mL per 100 g of preparation was standardized.

The data with household measurements from the 24HRs were converted to units of measurement (grams or milliliters) by the Table to Evaluate Food Consumption in Household Measurements [53] to calculate the number of servings.

The number of daily servings was adjusted by 1000 kcal/day. Thus, the scores for each food component and the BHEI-R total score were calculated. For most components, the recommendations by the Food Guide for the Brazilian Population [54] regarding the number of daily servings were considering when adopting the criteria for establishing the minimum, intermediate and maximum scores. For components such as saturated fat, sodium and SoFAAS, the higher the intake, the lower the assigned score. The maximum BHEI-R total score is 100 points. For each food component, the scores given are zero (minimum), 5, 10 or 20 (maximum), depending on the food group. Details on calculating the BHEI-R score can be found in Lima et al. [55]. To calculate the percentage of inadequacy of the BHEI-R components, the percentage of women who did not reach the maximum score for each one was analyzed. To assess the diet quality of each patient, stratification of the total score in tertiles was performed considering intervals equivalent to the baseline time (T0). Thus, the classification of the diet quality was made according to the following cut-off points at the three sessions of the study (T0, T1 and T2): “inadequate diet” for scores below 58.46; “diet requires modifications” for scores below 64.38; and “healthy diet” for scores equal to or greater than 64.38. A total BHEI-R score below 64.38 as a cardiovascular risk factor and that the adoption of a better-quality diet is associated with a reduced risk of mortality from CVDs were considered [16,17].

### 2.7. Cardiovascular Risk Factors

Two methods (analysis 1 and 2) were used to count the risk factors per participant at each time, with all risk factors having the same load (Table 1). In analysis 1, 20 risk factors were considered (CT with anthracycline; radiotherapy; DM; systemic arterial hypertension; smoking; excessive alcohol consumption; physical inactivity; overweight; abdominal adiposity assessed by the WHtR; inadequate consumption of total, saturated, polyunsaturated, monounsaturated and trans fats, cholesterol, fiber, omega 3, omega 6/omega 3 ratio, sodium and total BHEI-R score) counted at each session of the study (T0 = 89; T1 = 65; T2 = 38). In analysis 2, 25 risk factors were considered (all variables considered in analysis 1 as well as the variables of the lipid profile: total cholesterol, LDL, HDL, non-HDL cholesterol and TGs) counted at T1 (*n* = 65) and T2 (*n* = 38). T0 was not included in analysis 2, as there was no blood collection at this time (Table 1).

After counting risk factors for CVDs, the average number of risk factors at each time was verified. A percentage of women with a certain amount of inadequate dietary factors was also evaluated among the 11 counted at each time (T0: *n* = 89; T1: *n* = 65; T2: *n* = 38).

### 2.8. Statistical Analysis

A generalized mixed model (GMM) was used to compare the mean values of the variables over time, adjusting the models by age, income, education and AI usage duration. The lipid profile was adjusted by cholesterol-lowering medication. In addition, the GMM was used to verify the impact of NRF (analysis 1 and 2) and the number of inadequate dietary factors on serum CRP levels and phase angle. For prospective analyses, the 38 women who participated in the three study sessions were considered. For the following analyses of the average number of risk factors for CVDs and percentage of inadequacy of CVD risk factors and dietary factors, all women recruited in the study were considered (T0 = 89; T1 = 65; T2 = 38 in analysis 1 and 2).

All statistical analyses were performed using IBM SPSS Statistics version 21.0, considering confidence intervals (CIs) of 0.95 and *p* < 0.05. The figures referring to the number of inadequate dietary factors and the average number of risk factors for CVDs were created using Excel.

## 3. Results

### 3.1. Sample Characterization

Table 2 shows the sociodemographic and clinical characteristics of the 89 BC survivors undergoing AI treatment, considering the baseline time (T0). Most participants were submitted to the following cardiotoxic treatments: chemotherapy with anthracycline (59.6%) and radiotherapy (84.3%). Regarding the history of CVDs, 12 women (13.5%) presented one or more of the following diseases: 1.1% hypertensive heart disease, 1.1% ischemic heart disease, 2.2% congestive heart failure, 1.1% mitral valve prolapse, 2.2% coronary artery disease, 1.1% angina, 2.2% arrhythmia, 2.2% acute myocardial infarction, 1.1% stroke and 1.1% cardiac form of Chagas disease. In relation to medications, 57.3% of the women used medications for SAH (34.8% diuretics, 25.8% beta-blockers, 10.1% calcium channel antagonists, 9% angiotensin-converting enzyme inhibitors, 28.1% angiotensin II receptor antagonists, 1.1% direct vasodilators and 1.1% sympatholytic drugs), 20.2% used medications for DM (16.9% biguanides, 7.9% sulphonylureas and 3.4% insulin) and 20.2% used medications (statins) for dyslipidemias.

### 3.2. Analysis of Changes in Risk Factors for CVDs over Time

No significant difference between the three evaluation times was found when comparing the total BHEI-R score (T0 = 60.31; T1 = 61.33; T2 = 61.22 points, *p* = 0.888, Table 3). In the analysis of the BHEI-R components, there was a significant increase in the consumption of the Meat, Eggs and Legumes component between T0 and T1 (*p* = 0.008), identified by the post hoc comparison. There was also an increase in the consumption of the Oils component between T1 and T2 (*p* = 0.022). For the other components, no significant changes were detected (Table 3).

Regarding the quantitative analysis of nutrients, there was less energy consumption, total carbohydrates, sugars, omega 6, total and monounsaturated fats at T1 and T2 than at T0 (*p* = 0.002; *p* < 0.001; *p* = 0.004; *p* = 0.011; *p* = 0.001 and *p* < 0.011). There was also less consumption of polyunsaturated fats at T1 when compared to T0 (*p* = 0.010). At T2, there was a reduction in the consumption of saturated fats and cholesterol, as well as an increase in the consumption of sodium and omega 3 (*p* < 0.001; *p* = 0.019, *p* = 0.002, *p* = 0.006, respectively). In addition, there was a reduction in the consumption of proteins, trans fats and omega 3/6 ratio (*p* < 0.001, for all) across the three times (Table 3). In addition, a reduction in protein, trans fats and the ratio of omega 6 to omega 3 consumption was found (*p* < 0.001, for all) across the three times (Table 3). It was also found that the consumption of total fiber did not change over time (*p* = 0.212), but there was a reduction in soluble fiber at T2 when compared to T0 and T1 (*p* = 0.015) (Table 4).

No changes over time were observed in anthropometric and body composition data, but a smaller phase angle was observed at T0 when compared to T1 and T2 (*p* < 0.001). In addition, there was a reduction in LAP at T2 (*p* = 0.007). Regarding biochemical results, a reduction in total cholesterol, non-HDL cholesterol, LDL, VLDL and TGs (*p* = 0.011, *p* = 0.002, *p* = 0.020, *p* = 0.038, *p* = 0.014, respectively) was identified, as shown in T2 (Table 5).

### 3.3. Percentage of Inadequacy and Number of Risk Factors for CVDs

**Figure 2:**
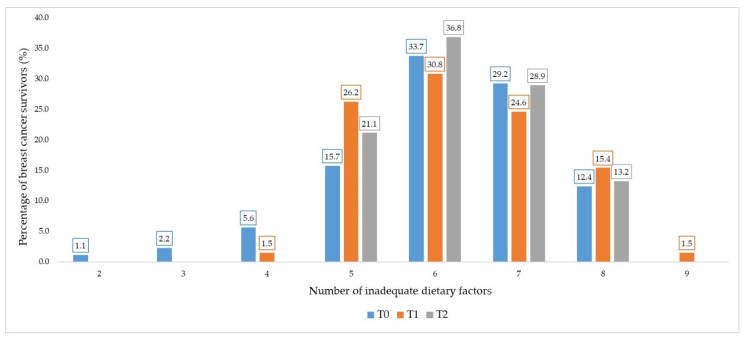
Percentage of breast cancer survivors with a certain number of inadequate dietary factors. Legend: eleven inadequate dietary factors were evaluated in each study period (T0, T1 and T2).

At the three sessions, a high percentage (>70%) of women did not reach the maximum score for the majority of BHEI-R components, except for the Oils component (T0 = 11.2%; T1 = 16.9%; T2 = 2.6%) (data not shown). The percentage of inadequacy of each risk factor for CVDs is shown in Table 6. A high percentage of inadequacy was observed for several nutrients: total fats (T0 = 77.5%; T1 = 98.5%; T2 = 92.1%), saturated fats (T0 = 96.6%; T1 = 100%; T2 = 100%), monounsaturated fats (100% at all times), fibers (T0 = 88.8%; T1 = 98.5%; T2 = 100%) and ratio of omega 6 to omega 3 (T0 = 95.5%; T1 = 98.5%; T2 = 92.1%). In contrast, cholesterol (T0 = 2.2%; T1 and T2 = 0%) and omega 3 consumption (T0 = 2.2%; T1 = 0%; T2 = 5.3%) showed low inadequacy (Table 6). In addition, the number of inadequate dietary factors was assessed at T0, T1 and T2. At all times, most women presented between six and seven inadequate dietary factors out of the 11 factors evaluated in the present study (Figure 2).

Regarding anthropometric data, there was a high percentage of overweight (T0 = 60.7%; T1 = 65.6%; T2 = 65.8%) and abdominal adiposity (WC > 80 cm: T0 = 85, 4%; T1 = 93.7%; T2 = 86.8%; WHtR ≥ 0.5: T0 = 93.3%; T1 = 93.7%; T2: 94.7%; WHR> 0.85: T0 = 68.5%; T1 = 71.4%; T2 = 68.4%) (Table 6).

As for the lipid profile, non-HDL showed a high percentage of inadequacy at T1 (43.4%). Women reported DM (T0 = 21.3%; T1 = 29.2%; T2 = 23.7%) and a high percentage of SAH (T0 = 56.2%; T1 = 58.5%; T2 = 55.3%) (Table 6).

For the lifestyle variables, a high percentage of physical inactivity was observed at all times (T0 = 59.6%; T1 = 47.7%; T0 = 57.9%). There was no excessive alcohol consumption in the women evaluated at any time (Table 6). At T0, T1 and T2, no woman had a complete absence of all risk factors for CVDs. Considering the 20 risk factors for CVDs (analysis 1), averages of 10.61, 10.78 and 10.84 factors were obtained at T0, T1 and T2, respectively. When the 25 risk factors for CVDs were assessed (analysis 2), there were averages of 11.63 and 11.37 factors at T1 and T2, respectively.

### 3.4. Number of Risk Factors, C-Reactive Protein and Phase Angle

The impact of the NRF (categorized by the median) on serum levels of CRP and PhA values was verified. Considering the 20 risk factors (analysis 1), it was observed that women with an NRF equal to or above the median (≥11 factors) had higher serum CRP levels than women with an NRF below the median (*p* = 0.003). There were no significant differences in the NRF categorization in the phase angle values, however, there was an increase in this variable at T1 and T2 in relation to T0 (*p* < 0.001) (Table 7). The analyses using the 25 risk factors (analysis 2) and the number of inappropriate dietary factors did not impact the CRP and PhA values.

## 4. Discussion

To the best of our knowledge, this is the first study to assess the number of risk factors for CVDs in BC survivors during endocrine therapy with AIs. It is important to evaluate aspects beyond cancer, to treat women in their entirety. The results of this prospective study indicate a high percentage of several risk factors for CVDs. Inadequacy of quantitative and qualitative food consumption was verified, with most of the evaluated nutrients not reaching the intake recommendations and important food groups of the BHEI-R had low scores. There was a high percentage of previous cardiotoxic treatments, excess body weight and abdominal adiposity, physical inactivity and the presence of comorbidities. Despite not having excessive alcohol consumption and having improved phase angle, LAP and lipid profile, with reduction in total cholesterol, non-HDL cholesterol, LDL, VLDL and triglycerides, most women had a worrying number of risk factors for CVDs at all times of the study. In addition, women with a higher NRF had higher levels of CRP, a predictor of cardiovascular risk. These observations are important to guide the multidisciplinary treatment for BC survivors.

In our study, we observed that most women underwent potentially cardiotoxic treatments, such as CT with anthracycline and radiotherapy. The literature shows that CT leads to inhibition of topoisomerase in both cancer cells and cardiomyocytes, causing cardiotoxicity due to the accumulation of double-stranded DNA breaks and mitochondrial dysfunction, which leads to the activation of cell death pathways and the accumulation of reactive oxygen species [56]. Radiotherapy, on the other hand, increases oxidative stress and inflammation, promoting a condition similar to atherosclerosis [57]. In addition to previous treatments, the current therapy with AIs may lead to increased cardiovascular risk [12]. As mentioned, this may occur due to a reduction in the levels of estrogen, a hormone associated with cardiovascular protection [13]. It is worth mentioning that, despite this, such treatments are extremely important for women diagnosed with BC [8] and should not be discouraged. Therefore, it is necessary to control modifiable factors related to CVDs such as diet, body composition, physical activity, smoking and alcohol consumption.

Despite the importance of controlling modifiable risk factors for CVDs, in the present study, there was a high percentage of inadequacy of both qualitative and quantitative dietary factors, overweight, abdominal adiposity and physical inactivity. Milliron et al. [58] showed that more than 90% of BC survivors did not meet the consumption recommendations for the Whole Fruit, Total Vegetables, Vegetables Dark Green and Orange Vegetables and Legumes, Whole Grains and SoFAAS components, similarly to the results of our study. Low adherence to a healthy diet can lead to negative health outcomes. A meta-analysis of prospective studies showed an inverse association between adherence to the Healthy Eating Index and Alternative Healthy Eating Index and the risk of all causes of mortality, including CVD mortality [59].

Despite the lower consumption of energy, carbohydrates, and sugars at T1 and T2, which suggests a reduction in food consumption, an overconsumption of total, monounsaturated and saturated fats was observed. In addition, the reduction in the consumption of polyunsaturated fats diverged from the recommendations for cardiovascular protection, which propose the substitution of saturated fats with polyunsaturated fats [60]. Furthermore, we found in the present study that the consumption of fibers and the omega 6 and 3 ratio did not meet the recommendations of the Brazilian Society of Cardiology [46]. Despite the low percentage of the inadequate consumption of cholesterol and omega 3, most women presented between five and six inappropriate dietary factors, among the 11 evaluated. Since quantitative and qualitative dietary aspects differ from the recommendations, it is advisable to implement protocols for dietary evaluation and intervention in cancer centers. Although there are quantitative nutritional recommendations for primary and secondary prevention of CVD, the emphasis of the latest guidelines has been, preferably, on qualitative counseling. In clinical practice, qualitative guidance is more easily understood, increasing the adherence [61].

Studies showed a high prevalence of physical inactivity among BC survivors [62,63], corroborating with our results. Possible determinants for physical inactivity in this population include old age, underweight and pain [64]. However, it is noteworthy that an increase in physical activity at any level is associated with a reduction in cardiovascular risk [65]. In addition, a study showed that adherence to physical activity recommendations was associated with better overall health and quality of life in BC survivors [66]. Due to the various benefits of physical activity, it is necessary to encourage this practice.

In addition to inappropriate diet and physical inactivity, there was a high frequency of overweight in our study. In patients with hormone-positive tumors, obesity may lead to BC-specific mortality [67] and is a cardiovascular risk factor by promoting inflammation, insulin resistance, endothelial dysfunction, coronary calcification and activation of coagulation, the renin–angiotensin system and sympathetic nervous system [68]. In addition, the prevalence of abdominal adiposity at all times in the present study is worrying, because even among women with weight within the recommendations, abdominal adiposity increases the risk of CVDs [69]. Women should be counseled on the importance of controlling body weight to reduce cardiovascular risk.

The conicity index (CI), visceral adiposity index (VAI) and LAP are important measures for assessing cardiovascular risk [40,44,45]. In the present study, we observed a reduction in LAP from T1 to T2, with no changes in CI and VAI. Considering that these measures do not have defined cut-off points, it is not possible to assess their excessiveness, requiring further studies. In the present study, there was no change in the fat-free mass and body fat percentage. A study of postmenopausal women showed that the percentage of fat and total lean mass are not predictors of cardiovascular risk, but high abdominal fat and reduced lower limb fat are relevant [70]. In clinical practice, monitoring waist circumference measurements throughout cancer treatment can be important for interventions to be established.

There was an increase in the phase angle at T1 and T2 in relation to T0. A study showed that women with BC who had a phase angle above 5.6 have a better prognosis [31]. Thus, the change in this measure can be considered positive, with a possible contribution to improving the prognosis of these women after T0. There was no association between the NRF and the phase angle in the present study. However, in a study with elderly subjects, the phase angle was associated with the global cardiovascular risk score, regardless of other factors [30]. Further studies are needed to focus on the clinical relevance of PhA in BC survivors and its impact on cardiovascular prognosis.

In the present study, comorbidities such as DM and SAH were reported, with the latter being observed in more than 50% of women at all evaluated times. BC survivors are more likely to have DM and systemic arterial hypertension than individuals not diagnosed with the disease [5]. These conditions, together with obesity, contribute to an increase in chronic inflammation, worsening the risk and prognosis of cancer, as well as increasing cardiovascular risk [71]. The adoption of a healthy lifestyle with the control of body weight, a healthy diet and physical activity is important for the prevention and treatment of chronic non-communicable diseases [72].

There was an absence of excessive alcohol consumption and a low percentage of smoking. A study showed that among survivors of various types of cancer, adherence to the recommendation of not smoking is more common than physical activity and the consumption of fruit and vegetables [73], corroborating our results. Further studies with a larger sample size are needed on the prevalence of smoking and alcoholism in BC survivors. However, the present study suggests that dietary interventions and physical activity are more urgent in this population.

Negative changes in the lipid profile increase cardiovascular risk, such as for atherosclerosis, stroke and heart attack [22]. However, in the present study, a reduction in total cholesterol, non-HDL cholesterol, LDL, VLDL and triglycerides between T1 and T2 was observed, without changes in other parameters. In addition, there was not a high percentage of inadequacy in the variables of the lipid profile, except for non-HDL cholesterol at T1 (51.3%). Such results contradict the possible negative effect of the use of AIs on the lipid profile [74].

Despite the absence of excessive alcohol consumption and positive changes in the lipid profile, the present study shows worrying results, with women presenting a higher average of risk factors for CVDs at all evaluated times. In women over 45 years of age, the greater the number of risk factors, the greater the mortality from CVDs. Compared with the absence of risk factors for CVDs, the presence of one, two or three or more raises the risk ratios for CVD mortality by 2.566, 3.655 and 5.416 times, respectively [75]. Considering that the women in the present study have a median age of 65 years, we emphasize the importance of assessing these factors in this population. Additionally, the women in the present study who were in the highest category of NRF had higher levels of CRP, an excellent predictor of cardiovascular events [28]. In addition to predicting cardiovascular risk, CRP plays important roles in the inflammatory process, including activation of the complement system, apoptosis, phagocytosis, release of nitric oxide (NO) and the production of cytokines, including interleukin-6 and tumor necrosis factor-α [27].

This study has some limitations. First, the biochemical data were collected only at T1 and T2, although the other variables were collected at the three evaluated times of the study. In addition, the assessment of food consumption is subject to error due to memory bias, but to minimize this, the interviews were conducted by properly trained nutritionists and the data were adjusted and mitigated to reduce inter- and intra-individual variability. A strength of the study was the evaluation of several variables, which allowed an overview of the cardiovascular risk profile of BC survivors. Furthermore, to the best of our knowledge, this is the first study to assess cardiovascular risk in BC survivors. Further studies, with larger samples, are needed to confirm our results.

Due to the high average number of risk factors for CVDs, the implementation of protocols to assess risk factors for CVDs should be suggested to women undergoing breast cancer treatment. In addition, lifestyle interventions to improve modifiable factors are of great value. A multidisciplinary approach is important, with dietary intervention, a physical training protocol, medical monitoring of pre-existing comorbidities and psychological monitoring to support behavioral changes. Psychological counseling is important for changing habits, and also for improving quality of life and reducing anxiety and depression in BC patients [76]. Another important aspect is the monitoring of cardiovascular health through specific tests during treatment and oncological follow-ups.

## 5. Conclusions

The results showed that most women had a worrying amount of risk factors for CVDs, and those with a higher NRF had higher levels of CRP, a predictor of cardiovascular risk. In the prospective analysis, an improvement in the lipid profile and phase angle was observed. Despite this, there was a high percentage of inadequacy in terms of dietary and anthropometric factors, in addition to the presence of comorbidities and physical inactivity. For this reason, emphasizing dietary and physical activity guidelines during and after treatment is necessary so that BC survivors can adopt healthier practices.

## Figures and Tables

**Table 1:** Risk factors for cardiovascular diseases counted per participant: analysis 1 and analysis 2.

Category	Factor	Criteria for Cardiovascular Risk	Analysis 1 (NRF = 20)	Analysis 2 (NRF = 25)
Treatment	Potentially Cardiotoxic Chemotherapy	Underwent	X	X
	Radiotherapy	Underwent	X	X
Comorbidities	Diabetes Mellitus	Presence	X	X
	Arterial Hypertension	Presence	X	X
Lifestyle	Smoking	Presence	X	X
	Alcohol Consumption	≥8 Drinks per Week	X	X
	Physical Activity	Physical Inactivity	X	X
Anthropometry	Overweight	Presence	X	X
	Abdominal Adiposity	WHtR > 0.5	X	X
Quantitative Food Consumption	Total Fat	>30% TCV	X	X
	Saturated Fat	>7% TCV	X	X
	Polyunsaturated Fat	<6% e > 10% TCV	X	X
	Monounsaturated Fat	<15% e > 20% TCV	X	X
	Trans Fat	>1% TCV	X	X
	Omega 3 Fatty Acid	<1 g/Day	X	X
	Omega 6/Omega 3 Ratio	>5:1	X	X
	Cholesterol	>300 mg/Day	X	X
	Fiber	Total < 25 g/Day and Soluble < 6 g/Day	X	X
	Sodium	<2300 mg/Day	X	X
Qualitative Food Consumption	Total BHEI-R	<64.38	X	X
Lipid Profile	Total Cholesterol	≥240 mg/dL	*	X
	Non-HDL Cholesterol	>160 mg/dL	*	X
	LDL Cholesterol	>160 mg/dL	*	X
	HDL Cholesterol	<40 mg/dL	*	X
	Triglycerides	>200 mg/dL	*	X

Analysis 1: T0, initial follow-up period; T1, intermediate period, corresponding to 12 months after T0; and T2, final follow-up period, corresponding to 24 months after T0. Analysis 2: T1 and T2. NRF, number of risk factors; BHEI-R, Brazilian Healthy Eating Index—Revised; LDL, low-density lipoprotein; HDL, high-density lipoprotein; WHtR, waist-to-height ratio; TCV, total caloric value. X, factors present in the analysis; * factors not available for analysis.

**Table 2:** Sociodemographic and clinical characteristics of breast cancer survivors at T0 (*n* = 89).

Variable	Median (p25–p75) and *n* (%)
Age (years)	65 (58.5–69.5)
Education level	
Below high school	61 (68.5)
High school or higher education	28 (31.5)
Income (minimum wage)	
<3	53 (59.6)
≥3	36 (40.4)
Surgery	
Breast-conserving surgery	51 (57.3)
Mastectomy	38 (42.7)
Radiotherapy	75 (84.3)
Chemotherapy	
Adjuvant	53 (59.6)
Neoadjuvant	15 (16.9)
Chemotherapy regimen	
Potentially cardiotoxic	53 (59.6)
Non-cardiotoxic	34 (38.2)
NR	2 (2.2)
Tumoral subtype	
Ductal	86 (96.6)
Lobular	3 (3.4)
Clinical stage	
I	26 (29.2)
II	48 (53.9)
III	13 (14.6)
NR	2 (2.2)
Tumor grade	
G1	14 (15.7)
G2	66 (74.2)
G3	5 (5.6)
NR	4 (4.5)
Positive estrogen receptor	85 (95.5)
Positive progesterone receptor	76 (85.4)
*HER-2* negative	71 (79.8)
Molecular subtype	
Luminal A	30 (33.7)
Luminal B	54 (60.7)
NR	5 (5.6)
Median AI usage time (in months)	29.5 (18.1–41.8)

T0: initial follow-up period; HER 2: *human epidermal growth factor receptor type 2*; NR: not reported; G1: well-differentiated tumor (low grade); G2: moderately differentiated tumor (intermediate grade); G3: poorly differentiated tumor (high grade); AI: aromatase inhibitor. The minimum wage was BRL 880.00.

**Table 3:** Brazilian Healthy Eating Index—Revised (BHEI-R) across T0, T1 and T2 (*n* = 38).

Component BHEI-R	Punctuation Minimum–Maximum	T0	T1	T2	*p*-Value
		Mean ± SE	Mean ± SD	Mean ± SD	
Total Fruit	0–5	2.81 ± 0.32	3.20 ± 0.26	3.04 ± 0.33	0.502
Whole Fruit	0–5	3.15 ± 0.36	3.42 ± 0.27	3.06 ± 0.39	0.410
Total Vegetables	0–5	3.46 ± 0.20	3.68 ± 0.17	3.82 ± 0.22	0.534
Dark Green and Orange Vegetables and Legumes	0–5	2.38 ± 0.27	2.87 ± 0.23	2.87 ± 0.28	0.318
Total Grains	0–5	4.43 ± 0.12	4.39 ± 0.10	4.31 ± 0.12	0.774
Whole Grains	0–5	0.70 ± 0.21	0.61 ± 0.17	0.89 ± 0.22	0.434
Milk and Dairy Products	0–10	4.73 ± 0.73	3.97 ± 0.64	4.08 ± 0.74	0.316
Meat, Eggs and Legumes	0–10	7.59 ± 0.35 ^a^	8.61 ± 0.24 ^b^	7.85 ± 0.32 ^a.b^	0.008
Oils	0–10	9.57 ± 0.18 ^a.b^	9.51 ± 0.18 ^a^	10.03 ± 0.13 ^b^	0.022
Saturated Fat	0–10	5.55 ± 0.50	5.73 ± 0.42	5.89 ± 0.52	0.913
Sodium	0–10	3.81 ± 0.42	3.26 ± 0.34	3.20 ± 0.44	0.444
Calories from SoFAAS	0–20	11.90 ± 1.02	12.22 ± 0.81	12.63 ± 1.06	0.884
Total BHEI-R	0–100	60.31 ± 2.15	61.33 ± 1.48	61.22 ± 1.88	0.888

T0, initial follow-up period; T1, intermediate period, corresponding to 12 months after T0; T2, final follow-up period, corresponding to 24 months after T0; SE, standard error; BHEI-R, Brazilian Healthy Eating Index—Revised; SoFAAS, calories from solid fats, alcohol and added sugars. A general mixed model (GMM) was used. Data adjusted for age, education, income and length of endocrine therapy with aromatase inhibitors. Post hoc comparisons: sequential Šidák. The different letters represent the differences between the times detected by the post hoc test. Results represented by the letter a differ from those represented by the letter b.

**Table 4:** Variation of energy and nutrients across T0, T1 and T2 (*n* = 38).

Nutrients	T0	T1	T2	*p*-Value
	Mean ± SE	Mean ± SE	Mean ± SE	
Energy (kcal)	1345.86 ± 46.31 ^a^	1161.59 ± 24.35 ^b^	1182.90 ± 22.30 ^b^	0.002
Protein (g)	60.21 ± 1.56 ^a^	54.54 ± 0.49 ^b^	51.50 ± 0.78 ^c^	<0.001
Carbohydrates (g)	173.90 ± 3.79 ^a^	150.44 ± 1.96 ^b^	153.60 ± 1.86 ^b^	<0.001
Sugars (g)	62.12 ± 3.44 ^a^	50.89 ± 1.97 ^b^	47.50 ± 2.34 ^b^	0.004
Total Fiber (g)	14.67 ± 0.53	14.07 ± 0.45	15.01 ± 0.55	0.212
Soluble Fiber (g)	3.83 ± 0.15 ^a^	3.59 ± 0.07 ^a^	3.35 ± 0.10 ^b^	0.015
Total Fat (g)	47.57 ± 1.01 ^a^	43.78 ± 0.48 ^b^	43.01 ± 0.71 ^b^	0.001
Saturated Fat (g)	15.45 ± 0.46 ^a^	15.34 ± 0.26 ^a^	13.52 ± 0.39 ^b^	<0.001
Polyunsaturated Fat (g)	11.40 ± 0.30 ^a^	10.40 ± 0.19 ^b^	10.56 ± 0.24 ^a.b^	0.010
Monounsaturated Fat (g)	16.38 ± 0.45 ^a^	14.27 ± 0.20 ^b^	14.32 ± 0.31 ^b^	<0.001
Trans Fat (g)	1.63 ± 0.07 ^a^	1.26 ± 0.04 ^b^	1.08 ± 0.05 ^c^	<0.001
Cholesterol (g)	185.53 ± 6.97 ^a^	175.91 ± 3.75 ^a^	151.19 ± 9.24 ^b^	0.019
Omega 3 Fatty Acid (g)	1.47 ± 0.03 ^a.b^	1.42 ± 0.03 ^a^	1.56 ± 0.03 ^b^	0.006
Omega 6 Fatty Acid (g)	9.82 ± 0.27 ^a^	8.90 ± 0.19 ^b^	8.87 ± 0.21 ^b^	0.011
Omega 6/Omega 3 Fatty Acid Ratio	6.77 ± 0.16 ^a^	6.30 ± 0.13 ^b^	5.70 ± 0.12 ^c^	<0.001
Sodium (mg)	2088.41 ± 53.65 ^a.b^	2060.03 ± 34.73 ^a^	2217.48 ± 45.00 ^b^	0.002

T0, initial follow-up period; T1, intermediate period, corresponding to 12 months after T0; T2, final follow-up period, corresponding to 24 months after T0; SE, standard error. A general mixed model (GMM) was used. Data adjusted for age, education, income and length of endocrine therapy with aromatase inhibitors. Post hoc comparisons: sequential Šidák. The different letters represent the differences between the times detected by the post hoc test. Results represented by the letter a differ from those represented by the letter b and c.

**Table 5:** Anthropometry, body composition and lipid profile across T0, T1 and T2 (*n* = 38).

Variables	T0 Mean ± SE; (*n* = X)	T1 Mean ± SE; (*n* = X)	T2 Mean ± SE; (*n* = X)	*p*-Value
BMI (Kg/m^2^)	28.56 ± 1.10 (*n* = 38)	29.29 ± 0.93 (*n* = 38)	29.45 ± 1.12 (*n* = 38)	0.308
WC (cm)	91.91 ± 2.95 (*n* = 38)	94.42 ± 2.32 (*n* = 38)	94.30 ± 3.07 (*n* = 38)	0.279
WHR	0.90 ± 0.02 (*n* = 38)	0.89 ± 0.02 (*n* = 38)	0.088 ± 0.20 (*n* = 38)	0.827
WHtR	0.59 ± 0.02 (*n* = 38)	0.61 ± 0.01 (*n* = 38)	0.61 ± 0.02 (*n* = 38)	0.284
FFM (Kg)	42.38 ± 1.08 (*n* = 29)	43.38 ± 0.87 (*n* = 28)	43.07 ± 1.15 (*n* = 27)	0.109
Body Fat (%)	40.55 ± 1.25 (*n* = 29)	39.36 ± 1.02 (*n* = 28)	39.78 ± 1.34 (*n* = 27)	0.367
PhA	5.40 ± 0.20 (*n* = 38) ^a^	6.27 ± 0.11 (*n* = 36) ^b^	6.11 ± 0.15 (*n* = 36) ^b^	<0.001
CI	1.27 ± 0.02 (*n* = 38)	1.29 ± 0.02 (*n* = 38)	1.28 ± 0.02 (*n* = 38)	0.560
LAP	-	71.34 ± 8.48 (*n* = 30)	47.40 ± 7.59 (*n* = 35)	0.007
VAI	-	2.70 ± 0.40 (*n* = 30)	2.12 ± 0.34 (*n* = 32)	0.102
Total Cholesterol (mg/dL)	-	199.20 ± 6.96 (*n* = 34)	179.37 ± 6.87 (*n* = 35)	0.011
Non-HDL Cholesterol (mg/dL)	-	152.00 ± 10.83 (*n* = 34)	129.35 ± 10.39 (*n* = 32)	0.002
LDL (mg/dL)	-	114.16 ± 5.83 (*n* = 33)	98.26 ± 5.27 (*n* = 33)	0.020
HDL (mg/dL)	-	52.76 ± 3.99 (*n* = 34)	54.69 ± 3.96 (*n* = 32)	0.565
VLDL (mg/dL)	-	32.13 ± 3.78 (*n* = 31)	26.80 ± 3.17 (*n* = 34)	0.038
Triglycerides (mg/dL)	-	164.30 ± 18.79 (*n* = 30)	133.65 ± 15.87 (*n* = 35)	0.014
CRP (mg/dL)	-	0.71 ± 0.14 (*n* = 33)	0.73 ± 0.15 (*n* = 34)	0.827

T0, initial follow-up period; T1, intermediate period, corresponding to 12 months after T0; T2, final follow-up period, corresponding to 24 months after T0; SE, standard error; BMI, body mass index; WC, waist circumference; WHR, waist-to-hip ratio; WHtR, waist-to-height ratio; FFM, fat-free mass; PhA, phase angle; CI, conicity index; LAP, lipid accumulation product; VAI, visceral adiposity index; LDL, low-density lipoprotein; HDL, high-density lipoprotein; VLDL, very low-density lipoprotein; CRP, C-reactive protein. A general mixed model (GMM) was used. Data adjusted for age, education, income and usage length of aromatase inhibitors. Total cholesterol and its fractions, triglycerides, LAP and VAI were also adjusted by cholesterol-lowering medication. Post hoc comparisons: sequential Šidák. The different letters represent the differences between the times detected by the post hoc test. Results represented by the letter a differ from those represented by the letter b.

**Table 6:** Percentage of inadequate risk factors for cardiovascular disease at T0, T1 and T2.

Cardiovascular Risk Factor	Criteria	T0 (*n* = 89)	T1 (*n* = 65)	T2 (*n* = 38)
Total Fat	>30% TCV	77.5	98.5	92.1
Saturated Fat	>7% TCV	96.6	100	100
Polyunsaturated Fat	<6% ou >10% TCV	11.2	3.1	2.6
Monounsaturated Fat	<15% ou >20% TCV	100	100	100
Trans Fat	>1% TCV	47.2	49.2	34.2
Cholesterol	>300 mg/Day	2.2	0	0
Fiber	<25 g Total Fiber <6 g Soluble Fiber	88.8	98.5	100
Sodium	>2300 mg/Day	28.1	24.6	34.2
Omega 3 Fatty Acid	<1 g/Day	2.2	0	5.3
Omega 6/Omega 3 Ratio	>5:1	95.5	98.5	92.1
Total BHEI-R	<64.38	66.3	58.5	73.7
Diabetes	Presence	21.3	29.2	23.7
Arterial Hypertension	Presence	56.2	58.5	55.3
Physical activity	Physical Inactivity	59.6	47.7	57.9
Smoking	Presence	10.1	10.8	7.9
Alcohol Consumption	≥8 Drinks per Week	0	0	0
			% (*n* = X)	% (*n* = X)
BMI	Overweight	60.7	65.6 (*n* = 63)	65.8 (*n* = 38)
WC	>80 cm	85.4	93.7 (*n* = 64)	86.8 (*n* = 38)
WHtR	≥0.5	93.3	93.7 (*n* = 64)	94.7 (*n* = 38)
WHR	>0.85	68.5	71.4 (*n* = 64)	68.4 (*n* = 38)
Total Cholesterol	≥240 mg/dL	*	17.9 (*n* = 56)	5.7 (*n* = 35)
LDL Cholesterol	>160 mg/dL	*	7.3 (*n* = 55)	0 (*n* = 33)
HDL Cholesterol	<40 mg/dL	*	18.5 (*n* = 54)	12.5 (*n* = 32)
Non-HDL Cholesterol	>160 mg/dL	*	43.4 (*n* = 53)	25 (*n* = 32)
Triglycerides	>150 mg/dL	*	15.4 (*n* = 52)	17.1 (*n* = 35)

T0, initial follow-up period; T1, intermediate period, corresponding to 12 months after T0; T2, final follow-up period, corresponding to 24 months after T0. BHEI-R, Brazilian Healthy Eating Index—Revised; BMI, body mass index; WC, waist circumference; WHtR, waist-to-height ratio; WHR, waist-to-hip ratio; LDL, low-density lipoprotein; HDL, high-density lipoprotein; TCV, total caloric value. * Variables not collected at T0.

**Table 7:** Impact of number of risk factors (NRF) on C-reactive protein and phase angle.

		NRF		Model Effects Tests		
	Study Time	< 11	≥ 11	Fixed Effects	Df	*p*-Value
	Mean ± SE	Mean ± SE	Mean ± SE			
C-Reactive Protein		0.37 ± 0.13	0.69 ± 0.11	NRF	1	0.033
T1	0.51 ± 0.11	0.37 ± 0.16	0.65 ± 0.13	Time	1	0.690
T2	0.56 ± 0.11	0.38 ± 0.16	0.74 ± 0.12	NRF × Time	1	0.708
Phase Angle		6.03 ± 0.14	5.85 ± 0.13			
T0	5.40 ± 0.20 ^a^	5.62 ± 0.26	5.18 ± 0.24	NRF	1	0.256
T1	6.26 ± 0.11 ^b^	6.22 ± 0.16	6.31 ± 0.14	Time	2	<0.001
T2	6.15 ± 0.15 ^b^	6.24 ± 0.20	6.06 ± 0.16	NRF × Time	2	0.234

T0, initial follow-up period; T1, intermediate period, corresponding to 12 months after T0; T2, final follow-up period, corresponding to 24 months after T0. SE, standard Error. NRF, number of risk factors. NRF categorized according to the median. A general mixed model (GMM) was used. Data adjusted for age, education, income and duration of use of aromatase inhibitors. Post hoc comparisons: sequential Šidák. The different letters represent the differences between the times detected by the post hoc test. Twenty risk factors were evaluated (analysis 1). Results represented by the letter a differ from those represented by the letter b.

## Data Availability

The data presented in this study are available on request from the corresponding author. The data are not publicly available due to privacy and ethical requirements.
